# Longitudinal Imaging of Injured Spinal Cord Myelin and White Matter with 3D Ultrashort Echo Time Magnetization Transfer (UTE-MT) and Diffusion MRI

**DOI:** 10.3390/jimaging10090213

**Published:** 2024-08-30

**Authors:** Qingbo Tang, Yajun Ma, Qun Cheng, Yuanshan Wu, Junyuan Chen, Jiang Du, Pengzhe Lu, Eric Y. Chang

**Affiliations:** 1 Research Service, Veterans Affairs San Diego Healthcare System, San Diego, CA 92161, USA; q1tang@health.ucsd.edu (Q.T.); q3cheng@health.ucsd.edu (Q.C.); yuw100@ucsd.edu (Y.W.); juc073@health.ucsd.edu (J.C.); jiangdu@health.ucsd.edu (J.D.); plu@health.ucsd.edu (P.L.); 2 Department of Radiology, University of California, San Diego, CA 92093, USA; yam013@health.ucsd.edu; 3 Department of Neuroscience, University of California, San Diego, CA 92093, USA; 4 Department of Bioengineering, University of California, San Diego, CA 92093, USA; 5 Department of Bone and Joint Surgery, The First Affiliated Hospital, Jinan University, Guangzhou 510632, China; 6 Radiology Service, Veterans Affairs San Diego Healthcare System, San Diego, CA 92161, USA

**Keywords:** spinal cord injury, magnetization transfer, diffusion, myelin, white matter

## Abstract

Quantitative MRI techniques could be helpful to noninvasively and longitudinally monitor dynamic changes in spinal cord white matter following injury, but imaging and postprocessing techniques in small animals remain lacking. Unilateral C5 hemisection lesions were created in a rat model, and ultrashort echo time magnetization transfer (UTE-MT) and diffusion-weighted sequences were used for imaging following injury. Magnetization transfer ratio (MTR) measurements and preferential diffusion along the longitudinal axis of the spinal cord were calculated as fractional anisotropy or an apparent diffusion coefficient ratio over transverse directions. The area of myelinated white matter was obtained by thresholding the spinal cord using mean MTR or diffusion ratio values from the contralesional side of the spinal cord. A decrease in white matter areas was observed on the ipsilesional side caudal to the lesions, which is consistent with known myelin and axonal changes following spinal cord injury. The myelinated white matter area obtained through the UTE-MT technique and the white matter area obtained through diffusion imaging techniques showed better performance to distinguish evolution after injury (AUCs > 0.94, *p* < 0.001) than the mean MTR (AUC = 0.74, *p* = 0.01) or ADC ratio (AUC = 0.68, *p* = 0.05) values themselves. Immunostaining for myelin basic protein (MBP) and neurofilament protein NF200 (NF200) showed atrophy and axonal degeneration, confirming the MRI results. These compositional and microstructural MRI techniques may be used to detect demyelination or remyelination in the spinal cord after spinal cord injury.

## 1. Introduction

Conventional MRI of the spinal cord using T1-weighted and T2-weighted sequences is essential for the diagnosis and surgical treatment of patients with spinal cord injury (SCI) in clinical practice. They can reveal the location and extent of the damage to the spinal cord and the surrounding tissues, facilitating decision-making in a timely and appropriate manner [1]. While important in the clinical management of acute injury, these MRI sequences provide little information about the evolving neurodegenerative changes of the adjacent and remote spinal cord segments. Furthermore, there is no information on neural plasticity involved in spinal cord injury recovery or following treatment, particularly dynamic myelin changes resulting from axonal degeneration of injured neurons, and remyelination arising from axonal sprouting of non-injured neurons [2].

In the central nervous system (CNS), myelin is a specialized, multilamellar structure sheathing axons, and is principally composed of lipids and myelin-associated proteins [3]. Non-water protons in myelin as well as protons in water tightly bound to myelin have very short T_2_s and are “invisible” when conventional clinical MR sequences are used [4,5]. One approach is to employ magnetization transfer (MT), which exploits cross-relaxation between myelin protons and tissue water to indirectly assess myelin [4,6,7,8,9,10]. Of note, when a protocol combining MT with an ultrashort echo time (UTE-MT) using an echo time (TE) of 76 µs was compared with a routine protocol combining MT with a longer TE of 3 ms, the magnetization transfer ratio (MTR) using the UTE-MT technique had a better correlation with the myelin amount determined by immunostaining of myelin basic protein (MBP) (r^2^ = 0.71 versus r^2^ = 0.48) [8]. UTE-MT, as a compositional MRI technique, complements the more widely used quantitative technique of diffusion-weighted imaging, which probes microstructure. Measures such as the axial diffusivity (AD or λ_||_), radial diffusivity (RD or λ_T_), fractional anisotropy (FA), mean diffusivity (MD), and apparent diffusion coefficient (ADC) derived from this technique have provided information about axon size, and white matter fiber orientation and density, and are sensitive to spinal cord injury [11,12,13,14,15,16,17].

The main aim of this work was to develop protocols that can noninvasively and longitudinally monitor dynamic changes in spinal cord white matter following injury or subsequent therapy with a reasonable resolution that can be practically achieved in intact rats using an MRI field strength that is widely available. Toward this goal, we adapted a UTE-MT sequence for the measurement of myelinated white matter, as well as a diffusion-weighted sequence to quantify white matter changes in the spinal cord following a unilateral C5 hemisection lesion. We chose hemisectioning as it is a well-characterized model that produces predictable myelin degeneration in the ipsilesional side of the spinal cord caudally, while largely sparing myelin in the intact contralesional side [18,19,20,21]. In addition, subsequent neuroplasticity such as the sprouting of non-injured axons from the intact side to the injury side has also been demonstrated with this model [22,23,24]. The expected dynamic change in white matter and myelin in particular makes it a suitable model for testing the sensitivity of different compositional and microstructural MRI techniques. Previous studies in large species [16,25] and limited studies in rodents [11,13,15] measured the mean values of MTR and diffusion matrices by manually drawing regions of interest (ROIs) on selective white matter; such analyses did not quantify total myelin changes and did not take account of injury-induced myelin atrophy. Therefore, we also aimed to quantify total white matter separately from grey matter; however, it was challenging to manually draw ROIs along the white matter and grey matter boundary due to the small size of the rodent spinal cord. We developed a simple thresholding method on MTR and diffusion maps to generate myelinated white matter and white matter areas, respectively, and demonstrate the utility of these measures in spinal cord caudal to the C5 hemisection lesion. 

## 2. Materials and Methods

### 2.1. Animal and Surgery

Right lateral C5 spinal cord hemisection lesions were performed in five 8-week-old female Fisher 344 rats, and two additional rats without injury were used as controls. To make lateral C5 hemisection lesions, animals were deeply anesthetized using an anesthesia cocktail containing ketamine (50 mg/kg), xylazine (2.6 mg/kg), and acepromazine (0.5 mg/kg). A 2 mm long right hemicord was removed using iridectomy scissors and microaspiration, with visual verification to ensure complete transection ventrally and laterally. 

Rats were imaged at 2, 8, 14, and 20 weeks post-injury. All imaging was performed on a 3-T MRI scanner (Bruker BioSpec 3-T, Billerica, MA, USA) using an 82 mm volume coil for transmission and a 30 mm surface coil for reception. Anesthesia was induced with 4% isoflurane and maintained for the duration of the imaging at 1–1.5%. Respiration was maintained between 40 and 60 respirations per minute, and body temperature was maintained at 37 °C using a water-heated pad. Total scanning time was less than 2 h. During the MRI, rats were positioned prone with their heads facing forward, aligning their spinal cords largely parallel to the B0 direction.

### 2.2. UTE-MT MRI and MTR Measurements

**Figure 1:**
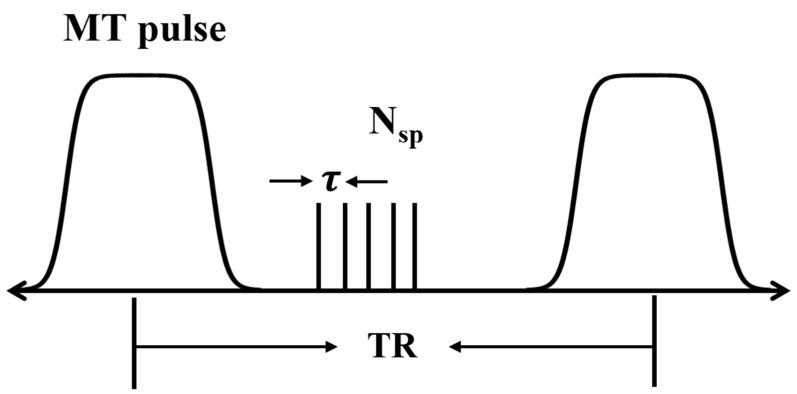
3D UTE-MT pulse sequence diagram. A Fermi-shaped pulse (duration =8 msec and bandwidth = 160 Hz) was used to generate MT contrast with three different flip angles of 1500°, 800°, and 0°, and a frequency offset of 1500 Hz.

A 3D UTE sequence was used with MT contrast obtained using a Fermi-shaped pulse (duration = 8 msec and bandwidth = 160 Hz) with three different flip angles (FAs) of 1500° (MT_1500), 800° (MT_800), and 0° (MT_0), and a frequency offset of 1500 Hz [26]. Figure 1 shows a sequence diagram of the 3D UTE-MT sequence. The detailed sequence parameters were as follows: TR/TE = 76/0.026 ms, field of view (FOV) = 24 × 24 × 84 mm^3^, matrix = 120 × 120 × 120, resolution = 0.2 × 0.2 × 0.7 mm^3^, number of spokes per-TR (N_sp_) = 9, spoke interval τ = 7.6 ms, FA = 10°, and bandwidth = 25 kHz, NEX = 3, with the total scan time of the sequence = 60 min. In addition, a product-stimulated echo-based B1 mapping sequence was also scanned to correct the B1 inhomogeneity of MTR measurement [27]. 

The MTR values were calculated on a pixel-by-pixel basis with MATLAB (The MathWorks Inc., Natick, MA, USA) using the following equations:

MTR_1500 = (MT_0 − MT_1500)/MT_0(1)

and

MTR_800 = (MT_0 − MT_800)/MT_0(2)

where MT_0, MT_800, and MT_1500 were the UTE signals with MT saturation flip angles of 0°, 800°, and 1500°, respectively.

B1-inhomogeneity correction was performed, and the final, corrected MTR map (MTR_corr) was used for data analysis [27]:

MTR_corr = (MTR_1500 + MTR_800)/2 + 1.64(1 − B1) x(MTR_1500 − MTR_800)(3)

Slices were analyzed 5 mm caudal to the injury or at corresponding locations for control rats. On the MTR maps, ROIs of the contralesional side of the spinal cord (including both grey and white matter) were drawn to obtain the mean MTR of the half section. The mean value was then used to threshold the MTR map to generate a myelinated white matter map. ROIs were also drawn on the injured half side of the spinal cord and the whole spinal cord, and were then thresholded with the mean MTR value from the contralesional side. Mean MTR values and total myelinated white matter areas were measured for the contralesional and ipsilesional sides, and corresponding locations for control rats. 

### 2.3. Diffusion-Weighted MRI and ADC Measurements

A fat-suppressed 2D diffusion-weighted echo-planar imaging sequence was used with diffusion encoding in three perpendicular gradient directions with b = 800 s/mm^2^. Images were acquired in the axial plane using the following parameters: TR/TE = 2300/22.8 ms, FOV = 28 × 28 mm^2^, matrix = 160 × 160, resolution = 0.175 × 0.175 × 3 mm^3^, and 12 slices, with the total scan time of the sequence = 8 min.

ADC values along the X, Y, and Z axes were calculated on a pixel-by-pixel basis (ADC_X, ADC_Y, and ADC_Z, respectively) with MATLAB using the following equation:

ADC = −ln (S/S_0_)/b (4)

where S is the signal intensity with diffusion gradients; S_0_ is the signal intensity without diffusion gradients (b = 0); and b is the b value.

FA maps were then calculated from the above values using the following equation:

FA = √(0.5 ×(√((ADC_X − ADC_Y)^2^ + (ADC_Y − ADC_Z)^2^ + (ADC_X –ADC_Z)^2^))/ 
√(ADC_X^2^ + ADC_Y^2^ + ADC_Z^2^)) (5)

We also calculated ADC ratio (ADC_R) maps, which reflect axial diffusivity normalized to radial diffusivity and were adapted from the literature [28], using the following equation:

ADC_R = 2 × ADC_Z/(ADC_X + ADC_Y)(6)

Slices were analyzed 5 mm caudal to the injury or at corresponding locations for control rats. On the ADC_R maps, ROIs of the contralesional side of the spinal cord (including both grey and white matter) were drawn to obtain the mean ADC_R of the half section. The mean value was then used to threshold the ADC_R map to generate a white matter map on the same slice. ROIs were also drawn on the injured half side of the spinal cord and the whole spinal cord, and were then thresholded with the mean ADC-R value from the contralesional half side. Mean FA and ADC_R values as well as total white matter areas were measured for the contralesional and the ipsilesional sides, and corresponding locations for control rats. 

### 2.4. Immunofluorescence Staining

Rats were transcardially perfused with saline (0.9%), followed by ice-cold 1X Z-fix. The spinal cords were then dissected out and post-fixed in 1X Z-fix overnight at 4 °C, cryoprotected with 30% sucrose, frozen, and cut at 50 µm. Sections were permeabilized and blocked with PBS containing 0.3% Triton X-100 and 5% donkey serum for 2 h; then incubated with mouse anti-NF200 (Millipore Sigma, Burlington, MA, USA, MAB5262, 1 µg/mL) and rabbit anti-MBP (Cell Signaling, Danvers, MA, USA, 78896, 1:1000) in PBS containing 0.1% triton X-100 and 5% donkey serum at room temperature overnight; washed for 1 h; then incubated with cy3-conjugated anti-mouse (Jackson ImmunoResearch, West Grove, PA, USA, Code 715-167-003, 1 µg/mL), DiLight649-conjugated anti-rabbit (Vector lab, Newark, CA, USA, DI1649, 1.5 µg/mL), and 0.2 µg/mL DAPI in the above solution for 4 h; washed with PBS; and then covered in PROTOS [29] with coverslips. Fluorescence images were taken with an inverted confocal laser scanning microscope (LSM880; Zeiss, Jenna, Germany) using a 10× objective. The following excitation lasers were used: DAPI, 405 nm; Cy 3, 568 nm; and Dylight 649, 633 nm. Fluorescence was collected based on the spectra of the fluorophores: DAPI, 415–460 nm; Cy3, 580–630 nm; and Dylight 649, >645 nm. 

### 2.5. Statistical Analysis

Differences in the means of the ipsilesional sides, contralesional sides, and controls for all time points and imaging measures were analyzed using a two-way ANOVA followed by Tukey’s multiple comparisons test. Receiver operating characteristic (ROC) curves were created for MRI measures, and area under the curves (AUCs) were calculated to determine the performance for distinguishing ipsilesional from contralesional sides. AUCs were interpreted as follows: 0–0.59, failed model discrimination; 0.6–0.69, poor; 0.7–0.79, fair; 0.8–0.89, good; and 0.9–1.0, excellent [30]. *p*-values less than 0.05 were considered to represent a statistically significant difference. Statistical analyses were performed with GraphPad Prism7 (Boston, MA, USA). 

## 3. Results

**Figure 2:**
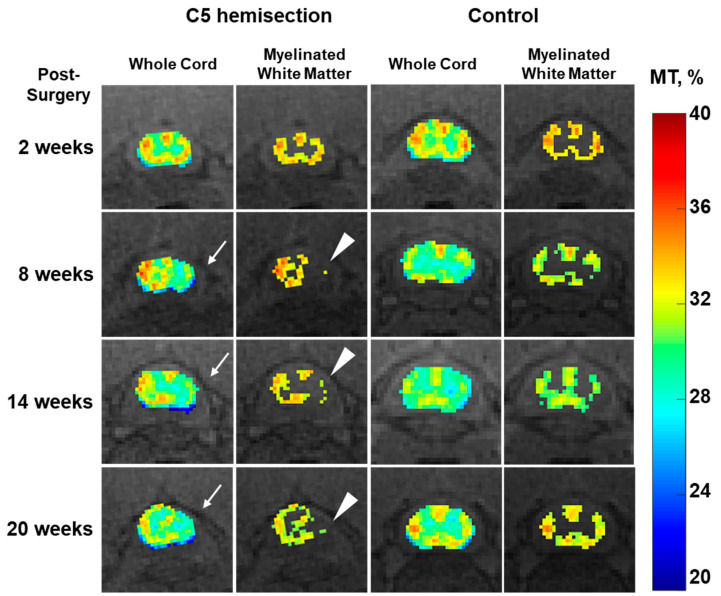
Longitudinal MTR mapping results of a representative rat at a location 5 mm caudal to unilateral C5 hemisectioning (columns 1 and 2) and the corresponding results from a control rat (columns 3 and 4). Decreases in MTR on the ipsilesional side are seen on the whole cord MTR maps of the injured rat beginning at 8 weeks (arrows). Myelinated white matter maps, which were obtained by thresholding using the mean MTR of the contralesional side on the same slice, show a markedly decreased total area on the injured side (arrowheads).

**Figure 3:**
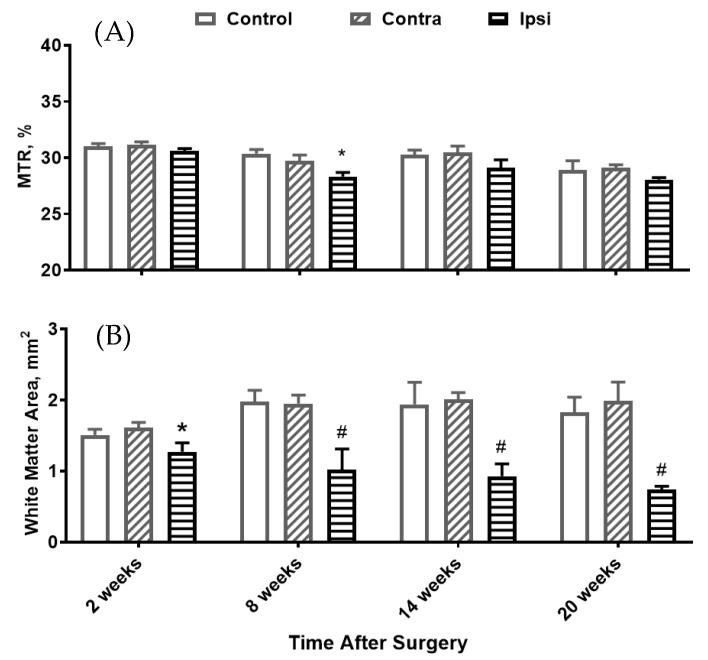
Time course of changes in MTR (**A**) and the myelinated white matter areas (**B**) of the contralesional side (contra), ipsilesional side (ipsi), and control rats at a location 5 mm caudal to a C5 hemisection. * *p* < 0.01, # *p* < 0.001 when the ipsilesional side was compared with the contralesional side using a two-way ANOVA followed by Tukey’s multiple comparisons test.

Representative MTR mapping results at a location 5 mm caudal to the C5 hemisectioning are shown in Figure 2. The mean MTRs of the ipsilesional side were decreased compared with that of the contralesional side beginning at 8 weeks, but the results did not reach significance (Figure 3A). Myelinated white matter regions have higher MTRs than grey matter regions, and they can be segmented by thresholding the MTR map using the mean MTR of the contralesional side of the same slice. The myelinated white matter areas were then obtained for the ipsilesional sides of the spinal cord, showing a dramatic decrease compared with that of the contralesional side that was statistically significant at all time points post-injury, even as early as 2 weeks (Figure 3B). 

**Figure 4:**
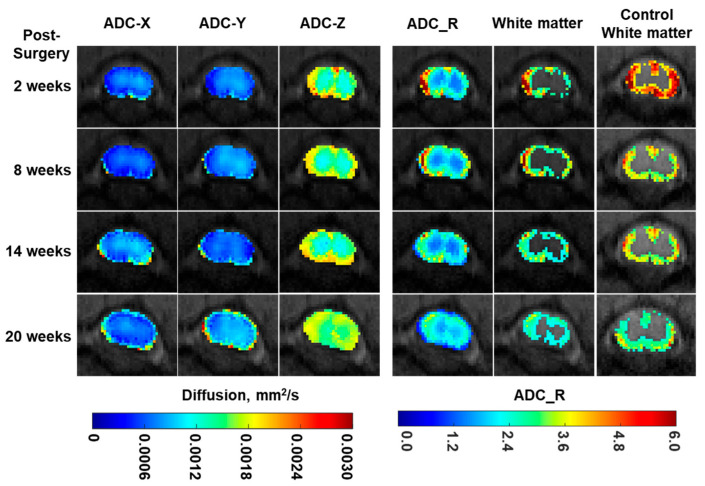
Longitudinal diffusion maps of a representative rat at a location 5 mm caudal to unilateral C5 hemisectioning in columns 1 to 5. Diffusivities in the radial (ADC_X and ADC_Y), axial (ADC_Z), and axial-over-radial diffusion directions (ADC_R) are shown as white matter maps, which were obtained by thresholding using the mean ADC_R value of the contralesional side on the same slice. The white matter maps of a control rat similarly obtained by thresholding are shown in the last column.

**Figure 5:**
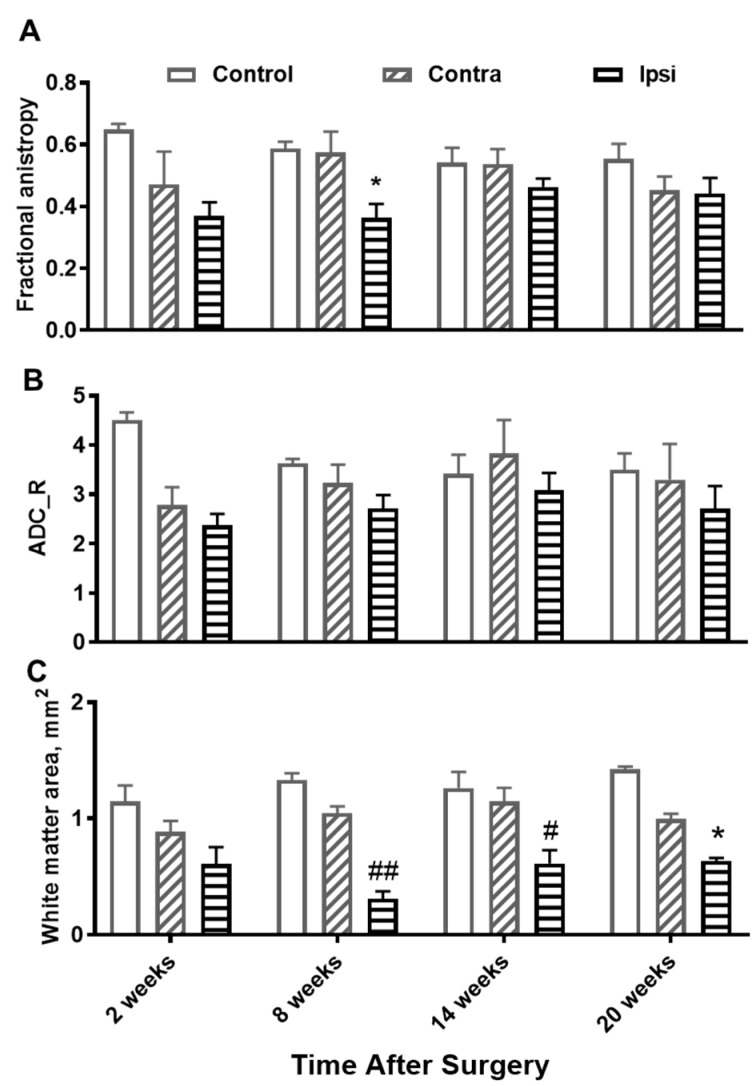
Time course of changes in fractional anisotropy (**A**), ADC_R (**B**), and the white matter area (**C**) of the contralesional side (contra), ipsilesional side (ipsi), and control rats at a location 5 mm caudal to a C5 hemisection. * *p* < 0.05, # *p* < 0.01, ## *p* < 0.0001 when the ipsilesional side was compared with the contralesional side with a two-way ANOVA followed by Tukey’s multiple comparisons test.

Diffusion-weighted MRI was also employed to image white matter changes following C5 hemisectioning. Figure 4 shows diffusion mapping depicting apparent diffusion coefficients along radial (ADC_X, ADC_Y) and axial (ADC_Z) directions. ADC_Z was consistently higher than ADC_X and ADC_Y, confirming that diffusion parallel to the axonal tracts in the spinal cord was greater than diffusion perpendicular to the axonal tracts. ADC_Z was also higher in white matter areas compared with grey matter areas. Differentiation between white versus grey matter was more pronounced when ADC_Z was normalized by the average of ADC_X and ADC_Y to obtain ADC_R. Using thresholding based on the mean ADC_R value of the contralesional side of the same slice, white matter areas can be segmented. At a location caudal to the injury, both fractional anisotropy and white matter areas of the ipsilesional sides were significantly lower compared with the contralesional sides beginning at 8 weeks (Figure 5). 

**Figure 6:**
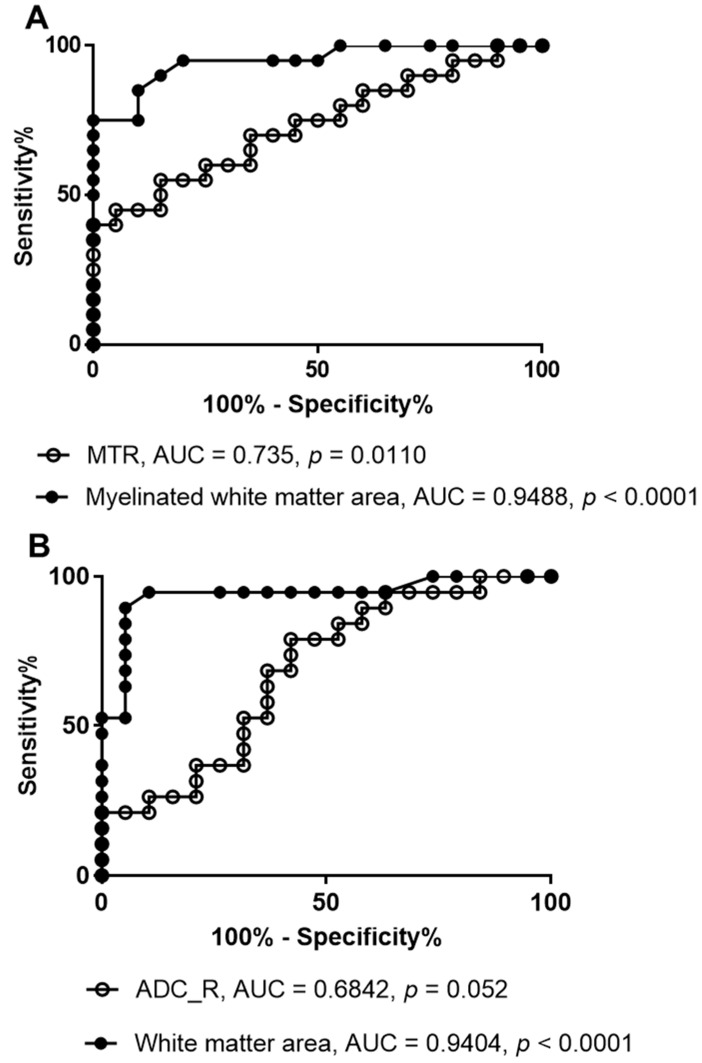
The receiver operator characteristic curve (ROC) analyses comparing MTR and the myelinated white matter area derived from thresholding of MTR (**A**), and comparing ADC_R and the white matter areas derived from thresholding of ADC_R (**B**). Data were from the contralesional and ipsilesional sides of five rats at week 2, 8, 14, and 20 post-injury.

Notably, the size of the myelinated white matter areas (obtained by thresholding the MTR maps) and white matter areas (obtained by thresholding the ADC_R maps) were more sensitive than the mean MTR or ADC_R values themselves for detecting changes after C5 hemisectioning. This was confirmed using ROC analysis where the performance for distinguishing ipsilesional from contralesional sides from the MTR values was fair (AUC = 0.74, *p* = 0.01) compared with excellent for the myelinated white matter areas (AUC = 0.95, *p* < 0.0001; Figure 6A). The discriminatory performance from the ADC_R values was poor (AUC = 0.68, *p* = 0.05) compared with excellent for the white matter area (AUC = 0.94, *p* < 0.0001; Figure 6B). 

**Figure 7:**
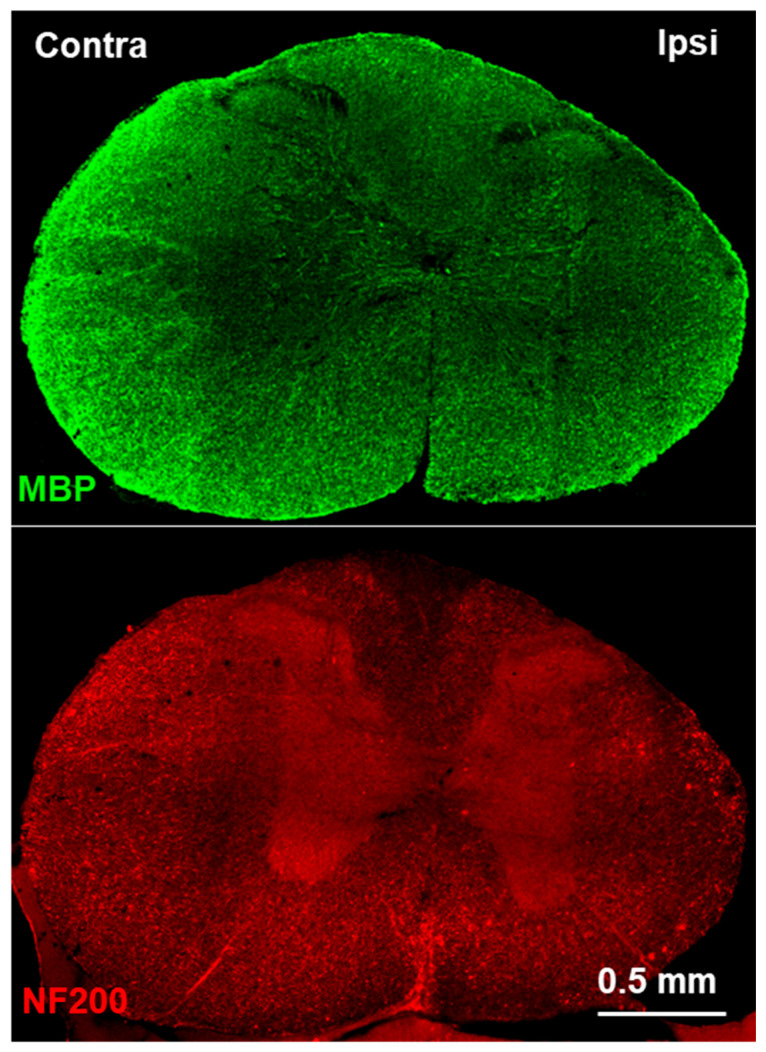
Immunofluorescence images after immunostaining for myelin basic protein (MBP, green, top) and NF200 (red, bottom) in a spinal cord section about 5 mm caudal to a unilateral C5 hemisection on the right side 20 weeks post-injury, showing atrophy and decreased staining intensity on the ipsilesional side.

Figure 7 confirms atrophy and decreased immunostaining intensity for MBP and NF200 in the ipsilesional side of the spinal cord caudal to the C5 hemisection. Atrophy was largely observed in white matter, which is consistent with the MRI results. In addition, there were some NF200-labeled structures in the ipsilesional white matter that were enlarged, resembling degenerating axon end bulbs [31]. 

## 4. Discussion

This study explored the uses of two complementary MRI techniques, namely magnetization transfer and diffusion, in detecting changes in myelin and white matter in the spinal cord caudal to a C5 hemisection. While both MTR values and diffusion parameters can differentiate white matter from grey matter, and some differences between the ipsilesional and the contralesional sides are seen, the parameters that are most sensitive in detecting the effect of the injury are the myelinated white matter areas obtained by thresholding the MTR and white matter areas obtained by thresholding the ADC_R maps. This is not surprising as MTR and ADC values are not white matter- or grey matter-specific, and any changes in caudal white matter caused by injury are expected to be masked by other spinal tissues that are largely spared in the spinal cord caudal to the injury. Thus, segmentation of white matter would increase the sensitivities of both techniques. However, it is a challenge to manually draw regions specifically for white matter and grey matter in the rat spinal cord due to the small size of the spinal cord, low resolution, and low contrast of these images. In addition, the difference in MTR values between white matter and the rest of the spinal tissues is rather small (confirmed in our unpublished results as well as in the literature [8,25,32]), thus limiting the dynamic range of this parameter for myelin detection. The difference in ADC_R between white matter and the rest of the spinal tissues is relatively larger, albeit more variable across different time points and among animals. This variability is likely due to subtle differences in cervical spinal cord positioning, the thickness of slices for imaging (3 mm along the *Z*-axis) as well as the limitation of a diffusion protocol that employed only three directions. Some of this variability can be reduced by increasing the in-plane resolution, decreasing slice thickness, and increasing the number of diffusion directions with diffusion tensor analysis, which is feasible considering that the current diffusion protocol only takes 8 min. 

We applied the mean of MTR or ADC_R of the contralesional side to threshold the MTR maps or ADC_R maps and successfully separated white matter from the rest of the spinal tissues. This approach is appropriate as histograms of both MTR and ADC_R of the contralesional sides are mostly nominal (Figure S1). It is similar to the approach that was used to separate white matter from grey matter and injured white matter using FA and radial and axial diffusivities [28], taking advantage of the fact that white matter has a higher axial diffusion and lower radial diffusion, which are reversed by injury [11,12,13,14,15,16,17,28]. This was a straightforward approach, but further refinements are possible based on the level of the slice to be analyzed. For example, it should be possible to calculate the proportion of the area occupied by grey matter at each level of the adult spinal cord, and then use the corresponding percentile of MTR or ADC_R from the contralesional side for thresholding. 

The longitudinal study also indicates that there is a trend for a continuing decrease in MTR and myelinated white matter in the spinal cord caudal to the injury, suggesting ongoing chronic demyelination after C5 hemisectioning. Indeed, at 20 weeks post-injury, degenerating axons are still observable. Interestingly, the decrease in ADC_R and FA appears to peak at week 2 or week 8, with a gradual recovery afterwards. The differences in these trends between UTE-MT-based measures, which probe myelin, and diffusion measures, which probe axonal microstructure, are promising and will be further explored in the future.

There are limitations to this study. First, a small sample size was used. However, this was a proof-of-concept study, and even with the small numbers, statistically significant differences and changes were observed. Second, for data processing and comparison, the intact, contralesional side of the same section was used as the reference. A caveat for this approach is that hemisectioning may potentially induce changes on the contralesional side. A comparison at the same axial slice may still be preferred as it could eliminate variation and inhomogeneity caused by the instrument, protocol, and positioning of the imaging coil and animals. Third, data-driven approaches for white matter segmentation such as using the median or a percentile of MTR and ADC_R values of the contralesional side to threshold the corresponding maps have not been exploited and compared in the current study. Finally, due to resolution limitations on a 3T system, the descending and ascending fiber tracts were not analyzed separately. Dramatic improvements in imaging time and resolution can be achieved through the use of higher field strength systems.

## 5. Conclusions

Dynamic changes in rats following a unilateral C5 hemisection lesion can be characterized with quantitative compositional and microstructural MRI techniques. The myelinated white matter area obtained through the UTE-MT technique and the white matter area obtained through diffusion imaging techniques show excellent performance to distinguish evolution after injury. These measures may have the potential to monitor the efficacy of stem cells or other remyelination treatments after spinal cord injury.

## Data Availability

Data will be made available on request.

## Supplementary Materials

Supporting information can be downloaded at https://www.mdpi.com/article/10.3390/jimaging10090213/s1. Figure S1: Examples of histograms of MTR (the left column) and ADC_R (the right column) of the contralesional intact half spinal cord of injury rats or the corresponding half spinal cord of control rats.
