# Supplementary material for: Longitudinal Imaging of Injured Spinal Cord Myelin and White Matter with 3D Ultrashort Echo Time Magnetization Transfer (UTE-MT) and Diffusion MRI
Source: J Imaging. 2024 Aug 30;10(9):213. doi: 10.3390/jimaging10090213 (PMC11433189; doi:10.3390/jimaging10090213)
Supplement: Supplementary file 1 [file jimaging-10-00213-s001.zip › jimaging-3129095-supplementary.pdf]

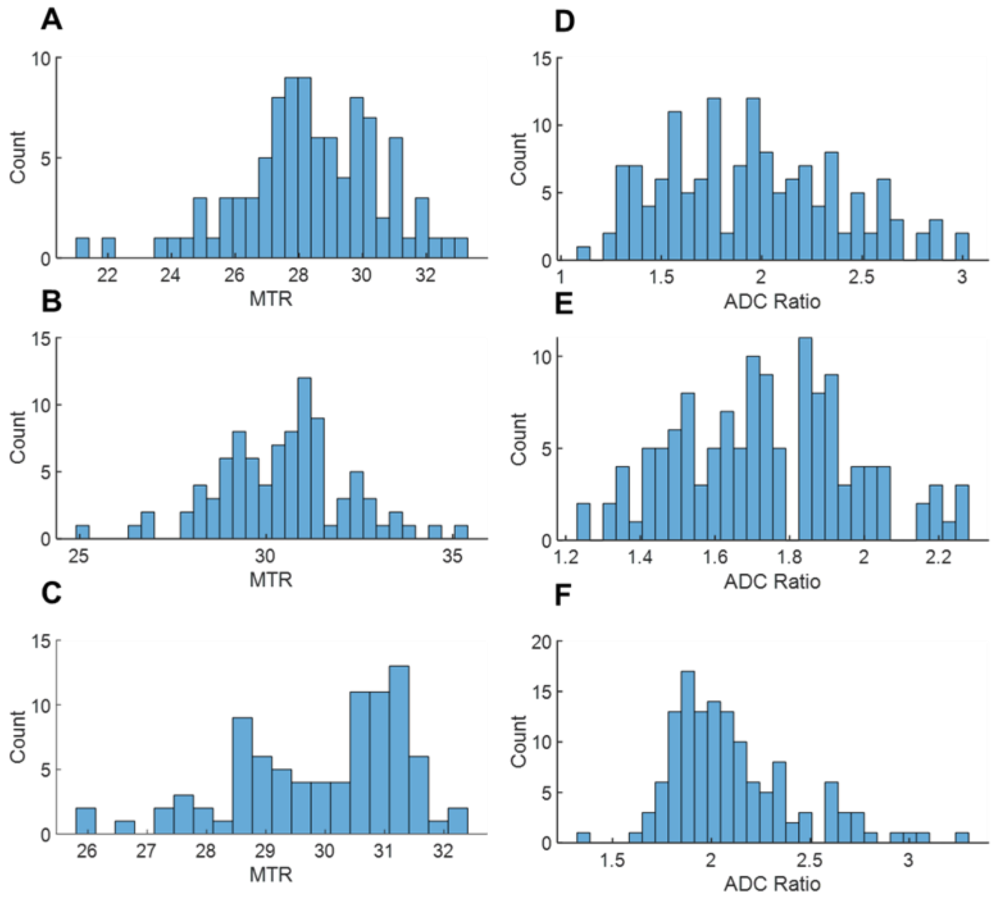

Figure S1. Examples of histograms of MTR (the left column) and ADC_R (the right column) of the contralesional intact half spinal cord of injury rats or the corresponding half spinal cord of control rats. Among all 20 histograms, eighteen passed (**A**, **B**, **D**, **E** are examples), and only two (**C**, **F**) failed the Kolmogorov–Smirnov test for nominal distribution
